# Local application of an ibandronate/collagen sponge improves femoral fracture healing in ovariectomized rats

**DOI:** 10.1371/journal.pone.0187683

**Published:** 2017-11-06

**Authors:** Jialiang Guo, Qi Zhang, Jia Li, Yansong Liu, Zhiyong Hou, Wei Chen, Lin Jin, Ye Tian, Linlin Ju, Bo Liu, Tianhua Dong, Fei Zhang, Yingze Zhang

**Affiliations:** 1 Department of Orthopaedic Surgery, the Third Hospital of Hebei Medical University, Shijiazhuang, P. R., China; 2 Key Laboratory of Orthopaedic Biomechanics of Hebei Province, Shijiazhuang, P. R., China; 3 Orthopaedic Research Institution of Hebei Province, Hebei, P. R., China; 4 VSD Medical Science & Technology Co., Ltd, Hubei, P. R., China; Mayo Clinic Minnesota, UNITED STATES

## Abstract

Non-union is a major clinical problem in the healing of fractures, especially in patients with osteoporosis. The systemic administration of drugs is time consuming and large doses are demanding and act slowly, whereas local release acts rapidly, increases the quality and quantity of the bone tissue. We hypothesize that local delivery demonstrates better therapeutic effects on an osteoporotic fracture. The aim of this paper is to investigate the effect of the local application of ibandronate loaded with a collagen sponge on regulating bone formation and remodeling in an osteoporotic rat model of fracture healing. We found that the local delivery of ibandronate exhibited excellent effects on improving the bone microarchitecture and suppressed effects on bone remodeling. At 4 weeks, more callus formation and improvement of mechanical character and microstructure were observed in a local delivery via μCT, mechanical test, histological research and serum analysis. The suppression of bone remodeling was compared with a systemic treatment at 12 weeks, and the structural mechanical properties and microarchitecture were also improved with local delivery. This research identifies an earlier, safer and integrated approach for local delivery of ibandronate with collagen and provides a better strategy for the treatment of osteoporotic fracture in rats.

## Introduction

Osteoporosis (OP) is a common disease that is characterized by the loss of bone mass, the deterioration of bone microstructure and increased fragility in elderly people, and it is classified as primary and secondary osteoporosis. Primary OP containing idiopathic osteoporosis is a common clinical disease, especially in postmenopausal women and elderly people, and the rate is higher as the life span of people is prolonged. Secondary osteoporosis indicates disease induced by drugs, such as glucocorticoids and heparin, or other diseases such as Huppert's Disease, diabetes, and Cushing Syndrome. OP may lead to fractures due to the weak strength of the bone. Rapid turnover of bone and no weight bearing of the fracture after OP may result in non-union, especially where osseous callus is scarce in the fracture site. Non-union of the osteoporotic fracture, which leads to loss of labor capacity and death, is the most commonly seen complication and results in a heavy burden for the family. The treatment of an osteoporotic fracture is difficult, and to minimize the negative result, it is essential to take active prevention and treatment as early as possible. Current therapies for non-union include surgical treatments and drugs. Surgical treatments can be conducted with iliacauto grafts, prosthesis or allografts with or without anabolic or catabolic reagents such as bone morphogenetic proteins (BMPs). Current therapies with drugs contain bisphosphonates, calcitonin, parathyroid hormone and calcium production.

Bisphosphonates (BPs) are compounds that are analogical with inorganic pyrophosphate and can inhibit bone resorption [1]. Bisphosphonates are classified as two categories (the non-nitrogen and nitrogen BPs) and act mainly through inhibiting the osteoclast attachment to bone matrix and inducing apoptosis action to regulate the balance between bone formation and resorption at the early stage of fracture healing [2]; they also affect the viability and migration ability of fibroblasts, vein endothelial cells and osteoblasts [3]. Ibandronate (IB) belongs to the third-generation nitrogen-containing BPs and is generated from the second generation by replacing hydrogen in the amino group with saturated hydrocarbon or the imidazole ring. It is used in the treatment of malignant tumors, skeletal metabolic disorders (Paget’s disease), osteoporosis or osteoporosis-related disease (lumbar intervertebral disc degeneration) and hypercalcemia of malignancy [4,5].

Oral or intravenous injections of IB are regular methods for the treatment of osteoporosis or fractures. The elimination of IB includes renal clearance and partitioning into bone (40%-50%) [5]. However, musculoskeletal pain, gastro-esophageal adverse events, osteonecrosis of the jaw and atypical fragility fractures associated with bisphosphonates are reported in the systemic administration of BPs [6]. Considering the negative effects and low bioavailability caused by systemic administration, local application of an ibandronate/gelatin sponge seems to be more preferable to improve healing of the fracture [7]. Local application of ibandronate is recognized as a promising method for the therapy of osteoporotic fractures and has gained attention in recent years [8,9].

A gelatin sponge was used for local delivery of IB in our previous study^7^. More drugs can be loaded because of its three-dimensional space maintainers and polyporous structures. Gelatin can be used as a carrier for local drug delivery, but it has no fixed structure and biological activity. Besides gelatin, oxidized cellulose and collagens are other local drug carrier materials, which have similar characteristics that include hemostatic and slow release [10]. Collagen (CS) is composed of three polypeptide chains intertwined with a typical right-handed triple-helix with a pitch of approximately 8.6 nm. With non-helical regions, it becomes a major structural protein responsible for the biomechanical properties of bone [11]. It is distributed in many organs and tissues, especially in connective tissues [10]. A total of 90% of extracellular protein of the tendon and more than 50% of the skin is composed of CS. As a biodegradable, biocompatible and nontoxic material, it promotes the proliferation of cells and accelerates the cohesion of platelets [10]. Because of its properties, biosynthetic CS provides the basis of a biomaterial and is used in various medical areas such as orthopedic and cardiac surgeries. Although there are many studies reporting the local application of BPs [7,12], few studies have investigated CS as a carrier and its effect on implant-bone integration [13]. The aim of this study is to investigate the hypothesis that local application of IB when combined with a collagen sponge (CS) as a delivery system can improve skeletal healing and mechanical stability compared with those with systemic administration in ovariectomized rats.

## Materials and methods

### Preparation of bovine collagen sponges combined with ibandronate

A fresh aqueous solution (7.8 mg/ml, pH 2–4, purity> 98.5%) containing typeⅠatelocollagen, which was generated from bovine tendon, was prepared from the BIOT Biology Company from Wuxi, Jiangsu Province in China. Sodium ibandronate at 1 mg/mL was purchased from the Biomedical Engineering Centre of Hebei Medical University in China. Collagen solution (3.9 mg/ml, 25 ml) diluted with pure water was loaded with IB (1 mg/mL), homogenized with a homogenizer at 20,000 rpm for 20 min, followed by crosslinking with glutaraldehyde (15 μl). The solution was added to a 1.5-cm mold and lyophilized at -50°C for 24 h. Moist tissue paper was placed on the surface to avoid collagen skin formation. The collagen sponge (10 mg) product consisted of collagen and 0.04 mg of IB and was sliced into units of 1.5 × 1.5 × 0.5 cm after being sterilized by Gamma rays for in vivo analysis. Collagens without IB in the Control group were also prepared with the same method. Our rationale for dose preference (0.004 mg, 0.04 mg and 0.4 mg) was based on previous studies, which have shown that the inhibition effect on osteoclasts as well as the callus property were more appropriate for bone regeneration with medium doses (0.04 mg) [14]. The collagen sponge was inserted laterally around the fracture site when the establishment of osteoporotic fracture was completed in rats.

**Fig 1:**
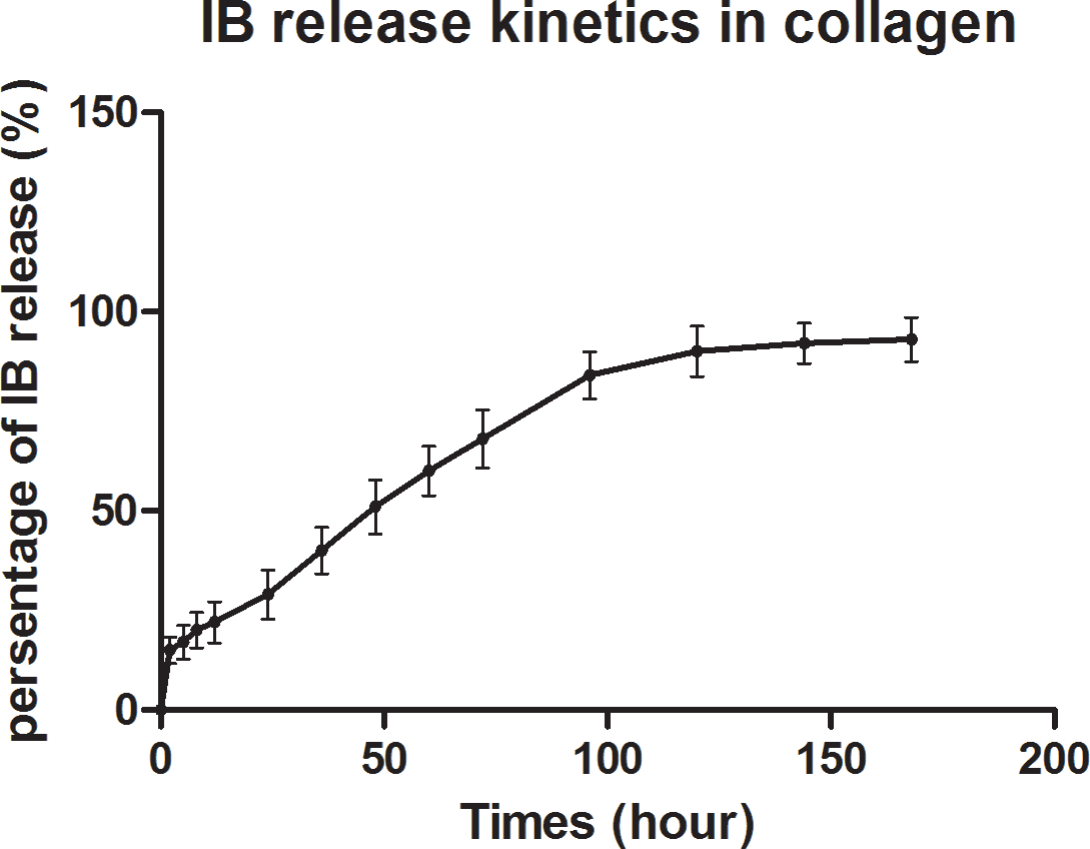
Quantification of IB release kinetics. The quantification of IB release kinetics from collagen in vitro (n = 6 for IB quantification at each time point).

For in vitro analysis of IB release kinetics, the collagen combined with IB was dispersed in the collagenase solution (0.8 mg/ml, 9001-12-1, Mp Biomedical, California, USA). It was placed in a shaking incubator in a 36°C environment. The supernatant was extracted at 0, 1, 2, 3, 4, 5 and 6 days post incubation. After each supernatant extraction, new collagenase solution (pH: 7.4) of equal volume was immediately supplemented. The released levels of IB were evaluated with high-performance liquid chromatography (Agilent 1100, Agilent Technologies, Santa Clara, California, USA) (Fig 1).

### Animal model and treatment

The animals were purchased from Hebei Medical University Animal Center. The study was approved by the Ethics Board of the Third Hospital of Hebei Medical University and was conducted in accordance with the institutional guidelines for the care and treatment of rats.

These rats were raised in a relatively stable ambient environment (temperature 24 ± 2°C, humidity 45% to 55% and 12-hour light-dark cycle from 7 a.m. to 7 p.m., specific pathogen-free SPF) with free access to tap water and rodent chow diet and were provided with nestlets and wood shavings for enrichment of the environment. Changes in appearance were monitored and addressed immediately. A total of 120 (24 animals per group) 12-week-old female Sprague–Dawley rats (300g ± 13g) were used in this study. All of the rats were operated on after a one-week acclimation period. Estrogen withdrawal was conducted by ovariectomy (OVX) via a dorsal approach in 96 SD rats, whereas 24 rats underwent sham surgeries in which the ovaries were simply exposed. The OVX rats were randomly divided into four groups (n = 24) by stratified randomization based on their baseline body weight.

At 12 weeks after OVX, the rats were all operated on to establish a femoral fracture model by middle lateral femoral fusion. Then, the rats without OVX were normally raised (Sham); rats received subcutaneous administration of vehicle (V = normal saline) in the Control group (OVX +V); CS without drug was locally applied in the CS group; and empty CS and subcutaneously injected (percutaneous injection, p. i) IB were used in the CS+IB group. The powder form of IB (1 mg/ml) was dissolved in 9 ml normal saline, and the concentration was adjusted to 0.1 mg/ml. The solution was administered subcutaneously in surgery as 0.015 mg/100 mg in the CS+IB (p. i) group. The others received local implantation of a collagen sponge containing IB in CS/IB. All of the rats were injected subcutaneously with 50 mg/kg tetracycline hydrochloride and 20 mg/kg calcein 12 days and 2 days before sacrifice, respectively.

### Surgical procedure

The rats were anesthetized using sodium pentobarbital through intraperitoneal injections (1%, 0.4 ml/100 g, Sigma-Aldrich, St Louis, MO, USA). The rats were then placed and fixed in a lateral position, and the fur overlying the right leg was shaved and sterilized. The operation was performed through a 1.0-cm medial lateral incision of the femur. The femoral shaft was exposed after stripping away the muscles. The traction to the incision skin can assist in exposing the femoral shaft, and a femoral osteotomy was performed using a wire saw (RA-II, 7.2V, Kangyu Medical Instruments Co., Ltd., Guangzhou, China). A sterilized Kirschner wire (ø1 mm, Jinhuan Medical Products Co., Ltd., Shanghai, China) was drilled into the proximal femoral canal and cut out from the proximal femur site. The traditional medullar insertion was used to fix the distal femur after the reduction of the osteotomy site, and the distal wire was buried in the surface of the joint. After flushing the osteotomy site, local implantations of sterilized collagen sponge with or without IB (units of 1.5 × 1.5 × 0.5 cm) around the osteotomy sites were performed laterally in the CS/IB, CS+IB and CS groups. The fracture lines were located in the middle shaft. The implants were rolled into a cylindrical shape and were implanted into the fracture site with a suture snare insertion device. Finally, the fascia and skin were closed. The rats received subcutaneous administration of vehicle (V = normal saline) in the Sham, Control and CS groups and 150 μg/kg IB in the CS+IB group as mentioned above. The rats were given prophylactic antibiotics (Penicillin-G; 200,000 U) soon after surgery for 3 days. The rats were permitted free activity after recovery from the anesthesia. Fractures that were not located in the midshaft femur were excluded.

X-ray images were obtained immediately after surgery to check the fracture alignment. All rats were fasted for 8 h before euthanization. The rats were anesthetized and euthanized by exsanguination via the abdominal aorta at the 4- and 12-week time points after conducting the femoral fracture model. The surface soft tissues and muscles were cleaned off prior to a series of structural and functional measurements. Half of the specimens were harvested, placed in individual tubes and then frozen at -20°C and prepared for Micro-CT scanning and biomechanical testing at each time point of euthanization. The others were kept in neutral buffered formalin (10%, pH 7.4) for histology. The specimens were decalcified with RapidCal Immuno (Lymed, USA), following dehydration steps using alcohol with different concentrations (70% to 100%) and xylene and were then embedded in paraffin. The sections were analyzed with a real-color image analyzing system (CBA 8000) at 200× magnification.

### X-ray photography and micro-CT scan

To observe bone formation and fracture union, anteroposterior X-rays (Carestream, digital DRX-1 System) were taken to evaluate callus formation and bridging bone formation at the fracture sites at 4 and 12 weeks with general anesthesia. Three observers independently graded the X-ray photography with a radiographic healing score system [15]. The three parameters contained gap size, bone in the gap, and mineralized callus. Each of them was assigned a score of 0, 1, or 2 with a composite score ranging from 0 to 6. The callus volume was identified around the osteotomy site. The osteotomized right femora were removed, and the relative soft tissues were dissected after the rats were euthanized. Femoral bones of rats in different groups were scanned using cone beam-type desktop micro-computed tomography (μCT40, Scanco Medical AG, Bassersdorf, Switzerland) with an isotropic voxel size of 10.5 μm (70 kV, 114 μA) and an acquisition of 500 projections per 180°. The noise of the gray-scale images was suppressed using a three-dimensional Gaussian filter with a protocol (sigma = 1.0 and support = 1.0). All samples were scanned within 2 days after sacrifice. Newly formed callus was differentiated from old cortices with established protocols (lower attenuation = 165 and upper attenuation = 368, μCT40 Evaluation Program v. 6.5–1; Scanco Medical). The tissues with the highest density represented highly mineralized new callus, whereas the low-density tissues represented newly formed callus. The region 5 mm proximal and distal (a total of 486 slices) to the osteotomy site was obtained as the region of interest (ROI). The ROI was chosen on the two-dimensional (2-D) CT images using manually drawn contours. The percent bone volume/total volume (BV/TV, %), trabecular thickness (Tb.Th, mm), trabecular number (Tb.N, 1/mm), trabecular separation (Tb.Sp, mm), and densities of BV and TV (mg HA/ccm) were calculated using the manufacturer’s software of the micro-CT machine. Multiplanar reformations were used to obtain three-dimensional images. A BMD threshold (211 mg/cm^3^) was defined to separate mineralized tissue from the surrounding substances.

### ELISA analysis of serum biomarkers of bone turnover

Serum marker levels were used as potential pathogenetic indicators of bone formation and bone resorption. Blood was collected (100 to 200 μL) from the abdominal aorta immediately prior to sacrifice. The sera and cells were centrifuged for 15 min at 5,500 RPM and 4°C and then stored at −80°C until assessment. After being thawed at two time points, serum levels were calculated with specific enzyme-linked immunosorbent assay (ELISA) kits for each animal. The effects of local implantation of the collagen sponge combined with IB were analyzed by CTX-I (cross-linked C-terminal telopeptide of type I collagen, which is the C-telopeptide degradation product released by type I collagen) and P1NP (procollagen type1 N-terminal propeptide). CTX-I is a biomarker for bone absorption and P1NP is used as a biomarker of bone formation, and they were quantified with commercial kits (Cusabio, Wuhan, China; intra- and interassay coefficients of variation [CV] for CTX were <7 and <8%, respectively; and intra-assay precision: CV <6%; inter-assay precision: CV <7% for PINP).

### Biomechanical test

The specimens were thawed overnight and immersed in 0.9% saline until testing at room temperature of 25°C. After the k-wires were removed, the specimens were bilaterally fixed. The testing machine with a maximal load of 225 N (BOSE 3520-AT, Eden Prairie, MN, USA) and built-in software was applied to assess the biomechanical properties with three bending tests to confirm the functional recovery. The rat femurs were horizontally positioned on the fixture (23 mm span) of the machine. The compression was located in the center of the osteotomy site at a speed of 2 mm/min and applied in the sagittal plane. The sagittal and coronal widths of the fractured femur were obtained using sliding calipers (Absolute 536, Mitutoyo, Japan). The measured parameters consist of maximum load and stiffness (obtained from the slope of the stress-strain curve), and the Young’s modulus and energy were derived from biomechanical tests.

### Histopathology

A 4% paraformaldehyde (Zaolutang Pharmaceutical Group Co., Ltd., Xi’an, China) PBS (pH 7.2–7.4) solution was used to fix the specimen for 2 days at 4°C. The femurs were decalcified for 8 weeks in RapidCal Immuno (Lymed, USA) at 4°C and processed for standard dehydration in graded alcohol and paraffin embedding. Sagittal 5-μm sections were cut (Microm HM360, Waldorf, Germany) from the osteotomy site and were then subjected to hematoxylin and eosin (HE) staining, Safranin O/light green-staining and Masson’s trichrome for histological evaluations according to standard methods. Histological images were acquired under light microscopy (BX51T-PHD-J11, Olympus, Japan) using an Axiocam ICc3 digital camera (Carl Zeiss) attached to the microscope and processed with built-in software.

The undecalcified histomorphometry was fixed in 4% paraformaldehyde (PFA), dehydrated, and cleared in xylene and then infiltrated and embedded in methylmethacrylate (MMA) without decalcification. A diamond saw (Leica SP1600, Leica Instruments, Nussloch, Germany) and hand grinder were used to section the femur longitudinally (~50 μm thick). Two repeated measurements were performed for each section within the selected ROI. For the histomorphometric analysis, the ROI was evaluated in stained slides covering 2.5 mm proximal and distal to the fracture line (total 5 mm). These undecalcified sections (by Stevenel's blue and Van Gieson's picrofuchsin for histomorphometric analysis) were performed with Image Pro software (version 5, Media Cybernetics, Inc., Silver Spring, MD) to quantify the trabecular bone histomorphometric parameters such as the callus area (Ca.Ar), lamellar/Ca.Ar, mineral apposition rate (MAR), and bone formation rate per bone surface (BFR/BS).

## Statistical analysis

The data were checked for normality and homogeneity of variance and analyzed using SPSS (version 21.0, SPSS Inc., Chicago, IL, USA). The results are presented as means ± standard deviation (SD). Frequencies are used to express the categorical data. The Kolmogorov-Smirnov test was used to examine the normal distribution, and Levene’s test was used for the homogeneity of variance. Analyses showed that all of the parameters obeyed normal distribution and homoscedasticity. Statistical differences between the Sham and Control groups were analyzed by the unpaired t-test. One-way analysis of variance (ANOVA) was applied with post hoc t-tests, and Tukey’s test was used to determine the statistically significant differences between the Control group and all other ovariectomized (OVX) groups. The significance level was set at 0.05.

## Results

### X-ray photography

Radiographs showed that the callus formed in the Sham group was high in density, partly union and similar to cortical bone at 4 weeks. In contrast, the fracture gap was clearly shown in the Control group. There was a greater amount of low density mineralized bone formation in the CS group compared with that of the Control, but bridging in the fracture gap was still scarce. Although newly formed bone was also low in density in the CS+IB and CS/IB groups, the mineralized callus was visually larger and bridging of the fracture ends with newly formed bone was faster than that in the Control and CS groups. The CS/IB group exhibited a significant increase in radiographic healing scores and fracture callus in comparison with the other three ovariectomized groups (more bridging was also observed).

**Fig 2:**
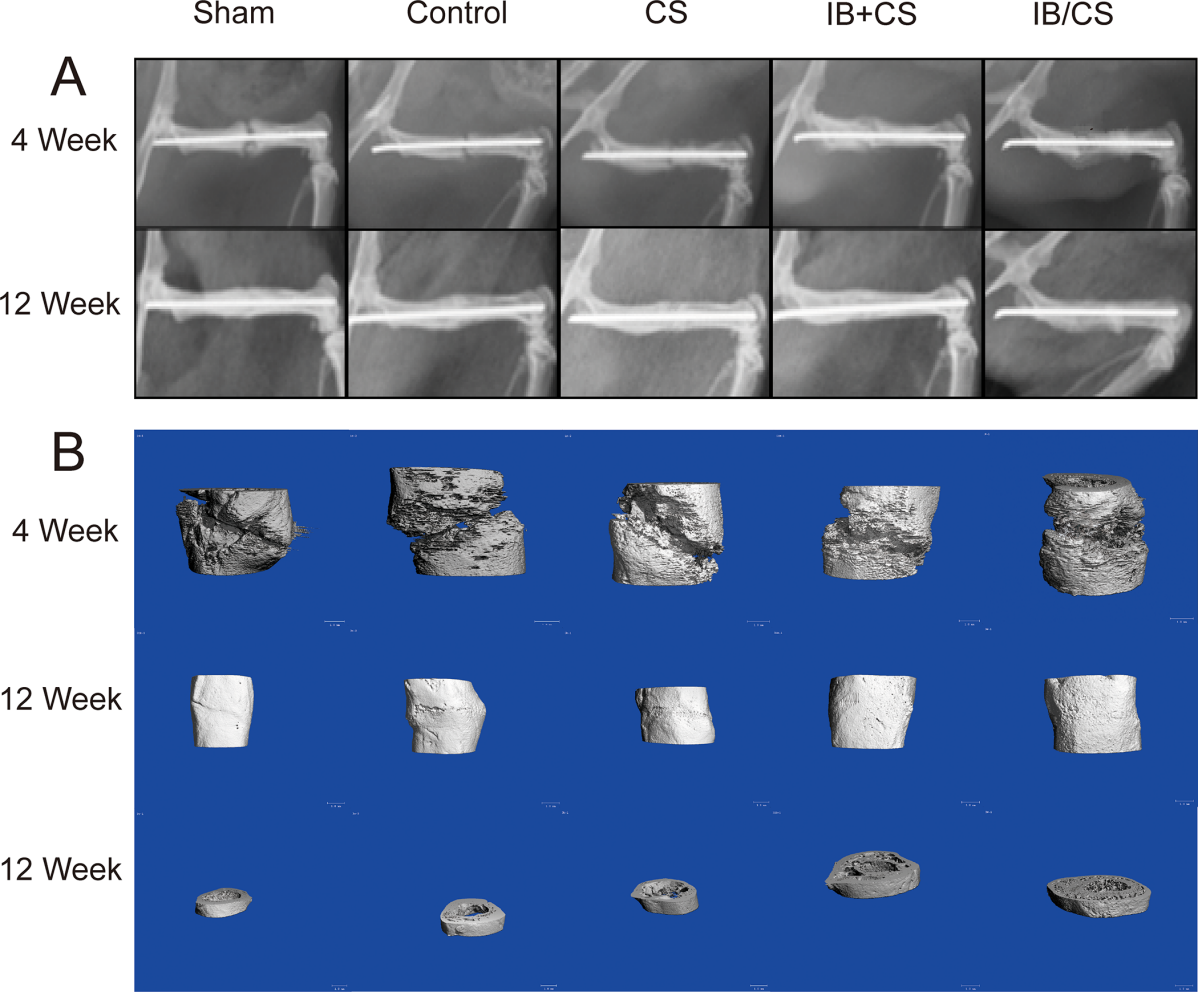
Results of local drug delivery (including CS, IB and their combination) into femoral fractures on bone union and microarchitecture in rats subjected to ovariectomy. (A) X-ray analysis. At 4 weeks, the callus formed in Sham was high in density, partly union and was similar to cortical bone. In contrast, the fracture gap was clearly shown in Control group. Twelve-week radiographs showed that the fractures of Sham group were united with remodeling completed, and the calluses of the IB-treated groups were much larger and lower density. (B) Three-dimensional reconstructed3-D Micro CT images in each treatment group. There was more mineralized callus forming bridge between fracture line in IB treatment compared with non-treatment group (Control and CS group) at 4 weeks. All groups entered into the remodeling phase at 12 weeks. Although the callus in the Sham and Control group all calcified to lamellar bone after remodeling, the osteoporotic status was obviously observed in Control. More external callus was shown in IB treatment groups (IB+CS and IB/CS) than that of CNT which means that the remodeling process was delayed. Sham: sham-operated group; Control: ovariectomized control group; CS group: local application of collagen sponge with ovariectomy; CS+IB group: local application of collagen sponge and systemic delivery of ibadronate with ovariectomy; CS/IB group: local delivery of collagen sponge combined with ibandronate.

**Fig 3:**
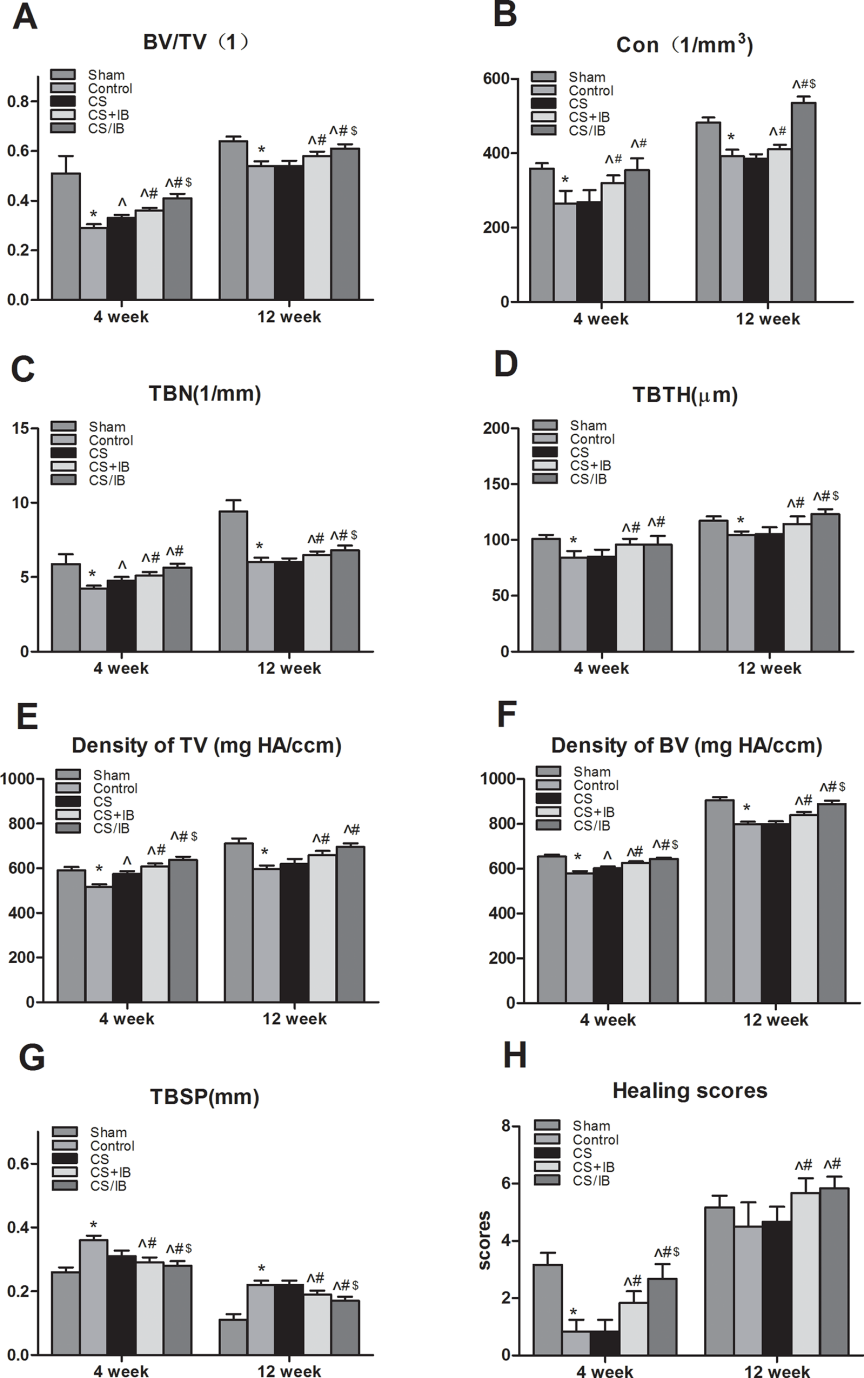
3D histomorphometry and fracture union scorings. (A-E) CS+IB and CS/IB groups at 4 weeks exhibited BV/TV density of BV and TV, Tb.Th, Tb.N and Conn.D were significantly increased and Tb.Sp reduced compared with those in Control group (p<0.05). The IB treatment group exhibited significant increase in BV/TV, density of BV, density of TV, Tb.Th, Tb.N and Conn.D, and lower in Tb.Sp than Control and CS group. The CS/IB also exhibited an improvement in trabecular bone parameter such as Tb.Th, Tb.N compared with CS+IB group. (F) There was significant improvement for CS/IB in scores compared with that in Control group at 12 weeks post injection. Values represent the mean ± standard deviation (SD). Each group consisted of 6 rats.*P< 0.05 compared to Sham by unpaired t-test; ^ P < 0.05 compared to Control; ^#^P < 0.05 compared to CS; ^$^< 0.05 compared to CS+IB. Tukey's multiple comparison test was applied were used to determine the differences between the Control group and all other ovariectomized (OVX) groups. CON, connectivity; TBN, trabecular number; TBTH, trabecular thickness; TBSP, trabecular separation.

At 12 weeks after fracture, bone union and an obvious reduction in callus size were observed in the Sham, Control and CS groups. A total of 9 of 12 fractures in the Control group and 11 of 12 rats in the CS group obtained a complete union at 12 weeks. The external callus was absorbed to restore the original cortical configurations in the Sham, Control and CS groups. Conversely, there was still some external callus that bridged to connect the end of the broken femur in the CS+IB and CS/IB groups. The callus size was not significantly larger in the CS/IB group than that of the CS+IB group (Fig 2A). There was a significant increase for CS/IB in the healing scores compared with that in the Control group 12 weeks after implantation (P < 0.05) (Fig 3H).

### Three-dimensional reconstructed images by μCT scans

Three-dimensional reconstructed μCT images in each group are shown in Fig 2.B. The histomorphometry of bone tissue around the fracture demonstrated that the callus with high density was obvious in the Sham group at 4 weeks. The CS group showed significantly increased Tb.N compared with that in the Control group. Consistent with X-ray radiographs, there was more mineralized callus bridged at the fracture site in the IB treatment groups (CS+IB and CS/IB groups) compared with the non-treatment groups (Control and CS groups). The CS+IB and CS/IB groups at 4 weeks exhibited BV/TV, Tb.Th, Tb.N, Conn.D, and density of the BV and TV that were significantly increased and a reduced Tb.Sp compared with those in the Control group (p<0.05). Furthermore, significant enhancement in the BV/TV and Tb.N was observed in the CS/IB in comparison with the CS+IB during the early period of fracture healing (P<0.05). There was a remarkable reduction of the Control group in the Tb.Th, Tb.N and Conn.D compared with those in the Sham group at 4 and 12 weeks.

No significant differences in bone microarchitecture were found between the CS and Control groups at 12 weeks. All groups entered into or completed the process of the remodeling phase. The CS+IB and CS/IB groups exhibited a significant increase in BV/TV, densities of BV and TV, Tb.Th, Tb. N and Conn.D, as well as lower Tb.Sp than the Control and CS groups. The CS/IB also exhibited an improvement in bone parameters such as Tb.Th and Tb. N compared with the CS+IB group (Fig 3).

### Serum levels of bone turnover biomarkers

**Fig 4:**
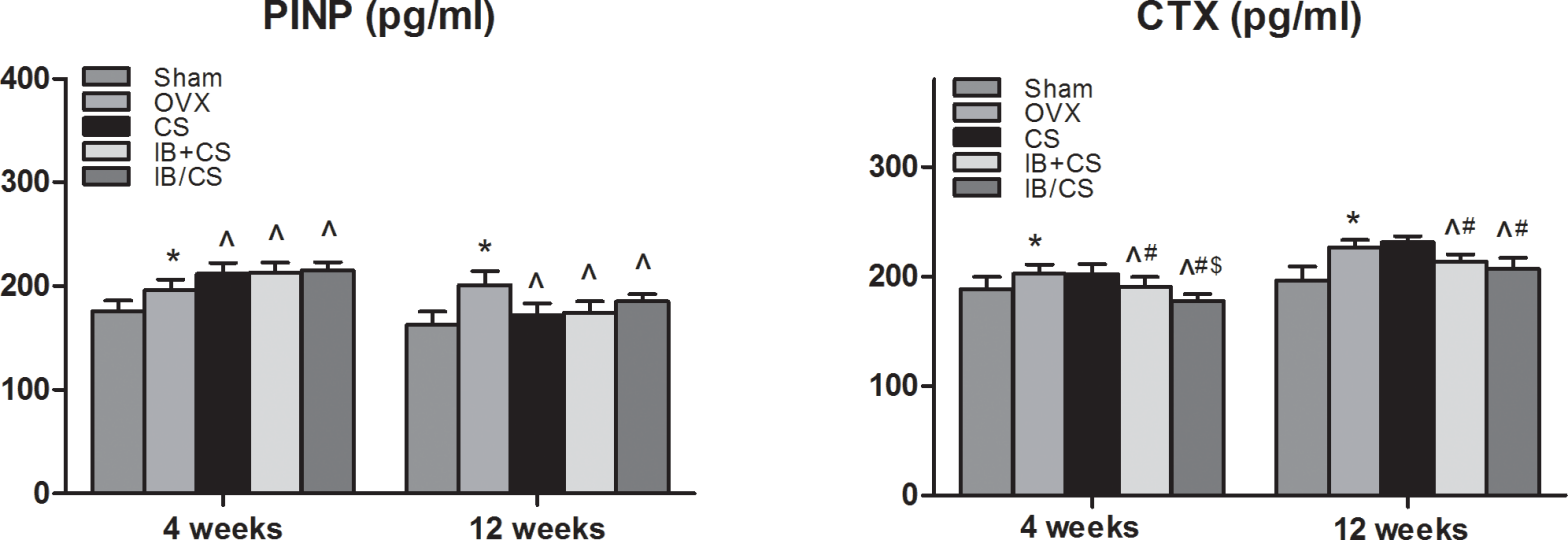
Serum levels of bone turnover markers. Serum biomarkers of bone formation, N-terminal propeptide of type I procollagen (PINP) and of bone resorption, C-terminal telopeptide of type I collagen (CTX-I) indicated that structural bone changes observed following injury are mainly caused by the decreased bone resorption with mild change in bone formation. Values represent the mean ± standard deviation (SD). Each group consisted of 6 rats.*P< 0.05 compared to Sham by unpaired t-test; ^ P < 0.05 compared to Control; ^#^P < 0.05 compared to CS; ^$^< 0.05 compared to CS+IB.

Compared with the Sham group, a significant increase in the serum level of the bone resorption marker (CTX) and the formation marker (PINP) was observed in the Control group. Consistent with the effect of IB, the CTX levels were significantly decreased in OVX rats supplemented with IB, especially in the CS/IB at 4 weeks. The overall average level of CTX in the CS+IB and CS/IB groups decreased to a level that was lower than that of the Control group. However, non-significantly decreased levels of CTX were illustrated at 12 weeks between CS+IB and CS/IB. There was a significant increase in the PINP level in the CS implanted groups (CS, CS+IB, and CS/IB groups) compared to the Control group at 4 weeks. Compared with the Control group, significantly elevated levels of PINP at 4 weeks following CS treatment indicate that CS application can improve the formation of callus, and the serum levels of PINP showed no significant changes in rats from the CS, CS+IB and CS/IB groups. At 12 weeks, the level of PINP decreased to a normalized level similar to that of the Control group, and no differences were found among those OVX rats in the four groups (Fig 4).

### Biomechanical test

To be concordant with our bone densitometry, the ultimate load in the Sham group was significantly higher than that of the Control group at 4 weeks. The IB treatment groups showed that the ultimate load was increased in comparison with that in the Control and CS groups. The energy parameters showed a significant increase in the IB treatment groups compared with the Control group, but there were no significant differences among the CS+IB and CS/IB groups. In terms of stiffness, there was an obvious improvement in CS/IB compared with that in other ovariectomized groups. Moreover, the IB treatment showed a significantly higher elastic modulus than the Control groups. There was no significant increase for CS/IB in the elastic modulus compared with that in the CS+IB groups. The Control group showed significant decreases in the ultimate load, energy stiffness and elastic modulus in comparison with those in the Sham at 4 and 12 weeks.

**Fig 5:**
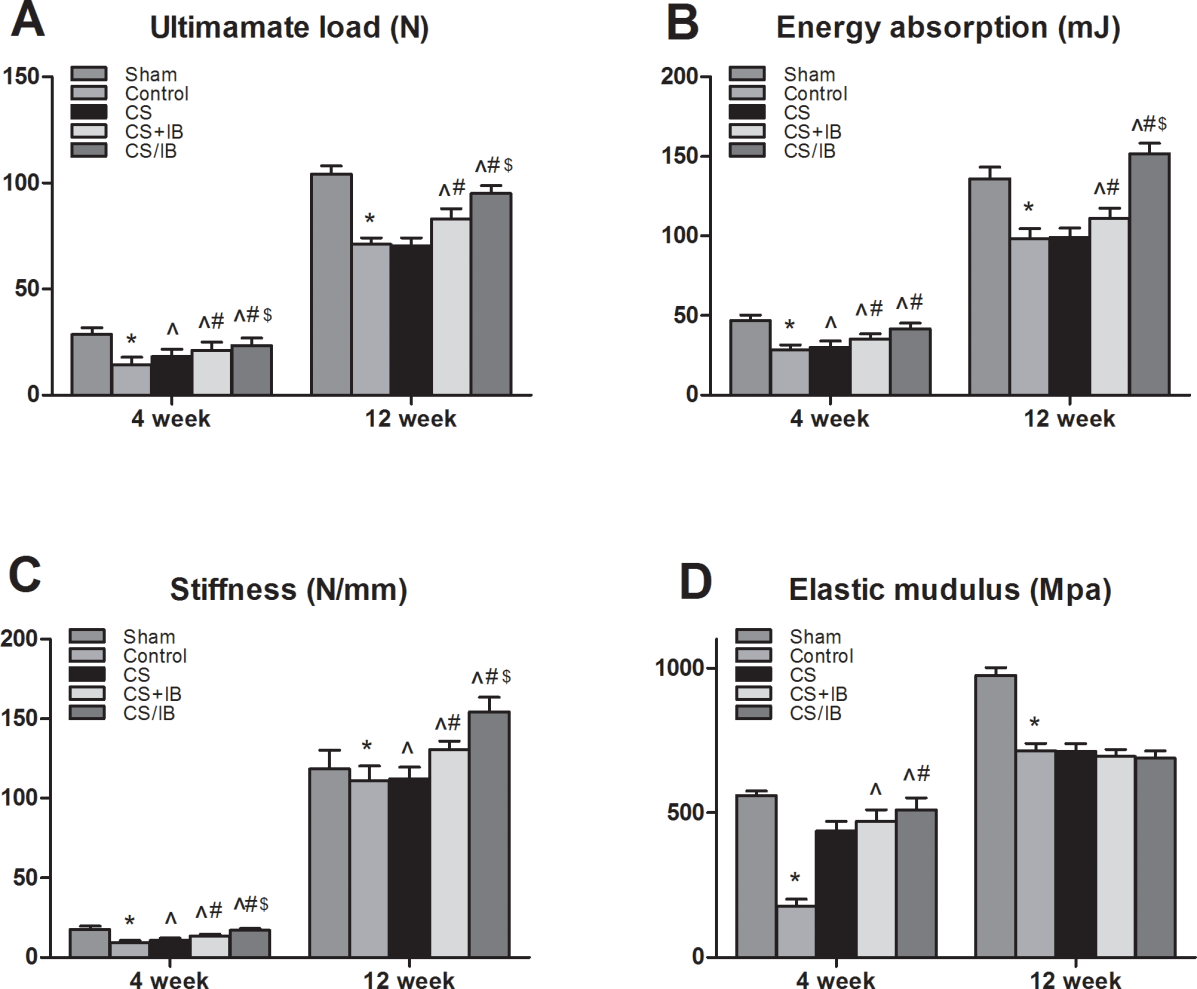
Combination treatment with IB plus CS increased femoral fracture strength. (A-C) The IB treatment group showed that mechanical parameters were increased in comparison with that in Control group. (D) No significant decrease for CS/IB in elastic modulus compared with that in Control, CS and CS+IB groups at 12 weeks. Values represent the mean ± standard deviation (SD). *P< 0.05 compared to Sham by unpaired t-test; ^ P < 0.05 compared to Control; ^#^P < 0.05 compared to CS; ^$^< 0.05 compared to CS+IB.

At 12 weeks, the mechanical parameters in the Sham group were significantly higher than the Control group. The IB treatment groups showed that the mechanical parameters were increased in comparison with that in the Control and CS groups. The ultimate load, energy absorbed and stiffness in the CS/IB treatment were highest compared with the other three ovariectomized groups. There was no significant decrease for CS/IB in the elastic modulus compared with that in the Control, CS and CS+IB groups (Fig 5).

### HE staining

The images of histological sections around the fracture site illustrated that the active endochondral ossifications and most of the process were completed in the Sham group at 4 weeks. A certain amount of cortical bone structure was also restored. However, the callus predominantly consisted of fibrocartilage, and sparsely distributed woven bone or remodeling of the cortex was formed in the Control group. The CS group showed an increased level of mineralization compared with the Control group, which indicated the osteoconductive property of the composite scaffolds. More woven bone with a random orientation of collagen fiber was observed in the IB treatment groups compared with the un-treated groups around the fracture line in OVX rats. The callus in the IB treatment groups still contains a large amount of fibrocartilage callus, which was in the stage of soft callus remodeling. Among the IB treatment group, the amount of woven bone in the CS/IB was larger than that of the CS+IB. The collagen sponge was found to be absorbed completely at 4 weeks in all five groups.

**Fig 6:**
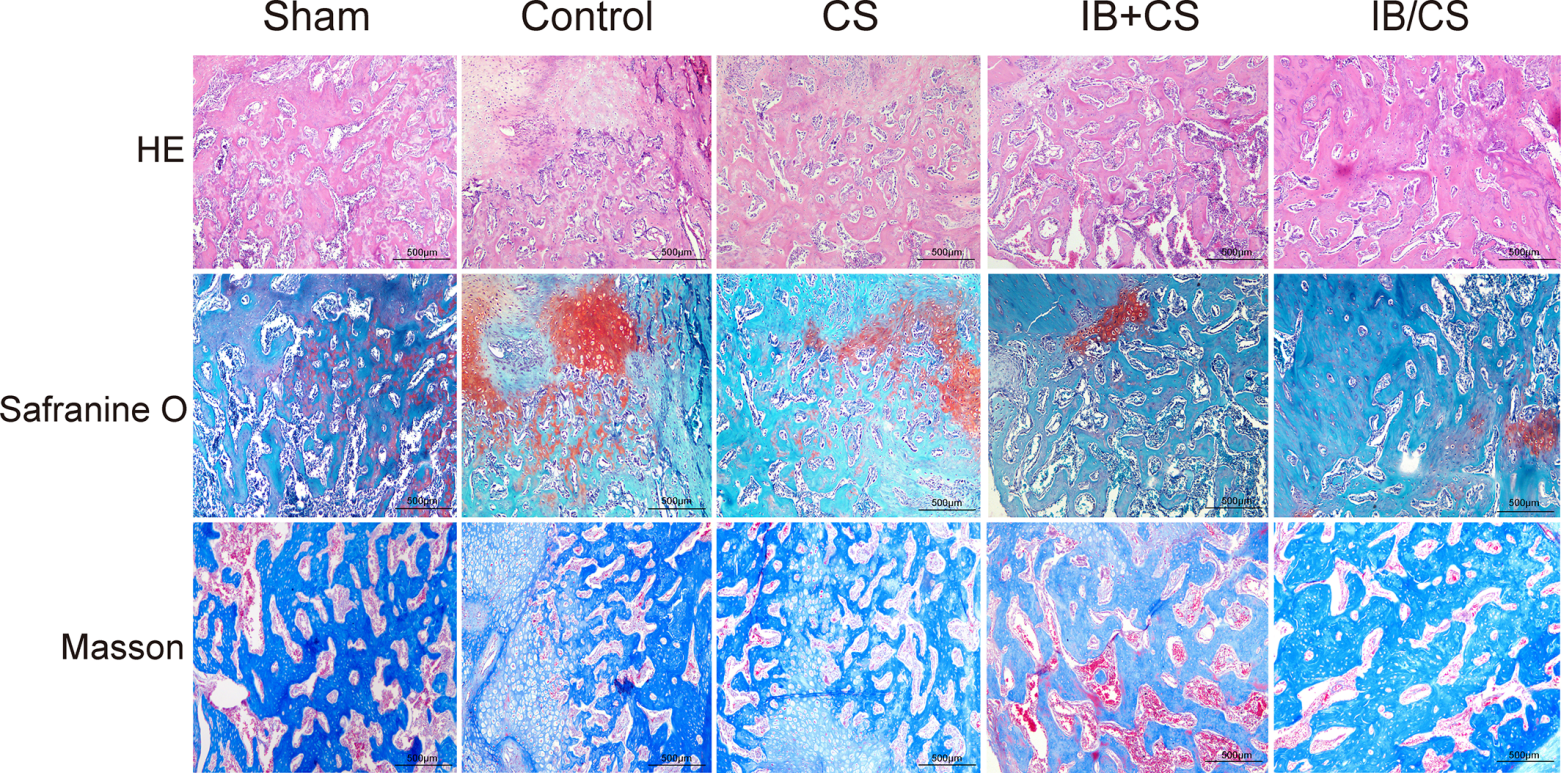
Histological evaluation of the fracture in each group at 4 weeks after surgery. HE staining, Safranin O and fast green staining, Masson staining. Scale bar = 500μm.

**Fig 7:**
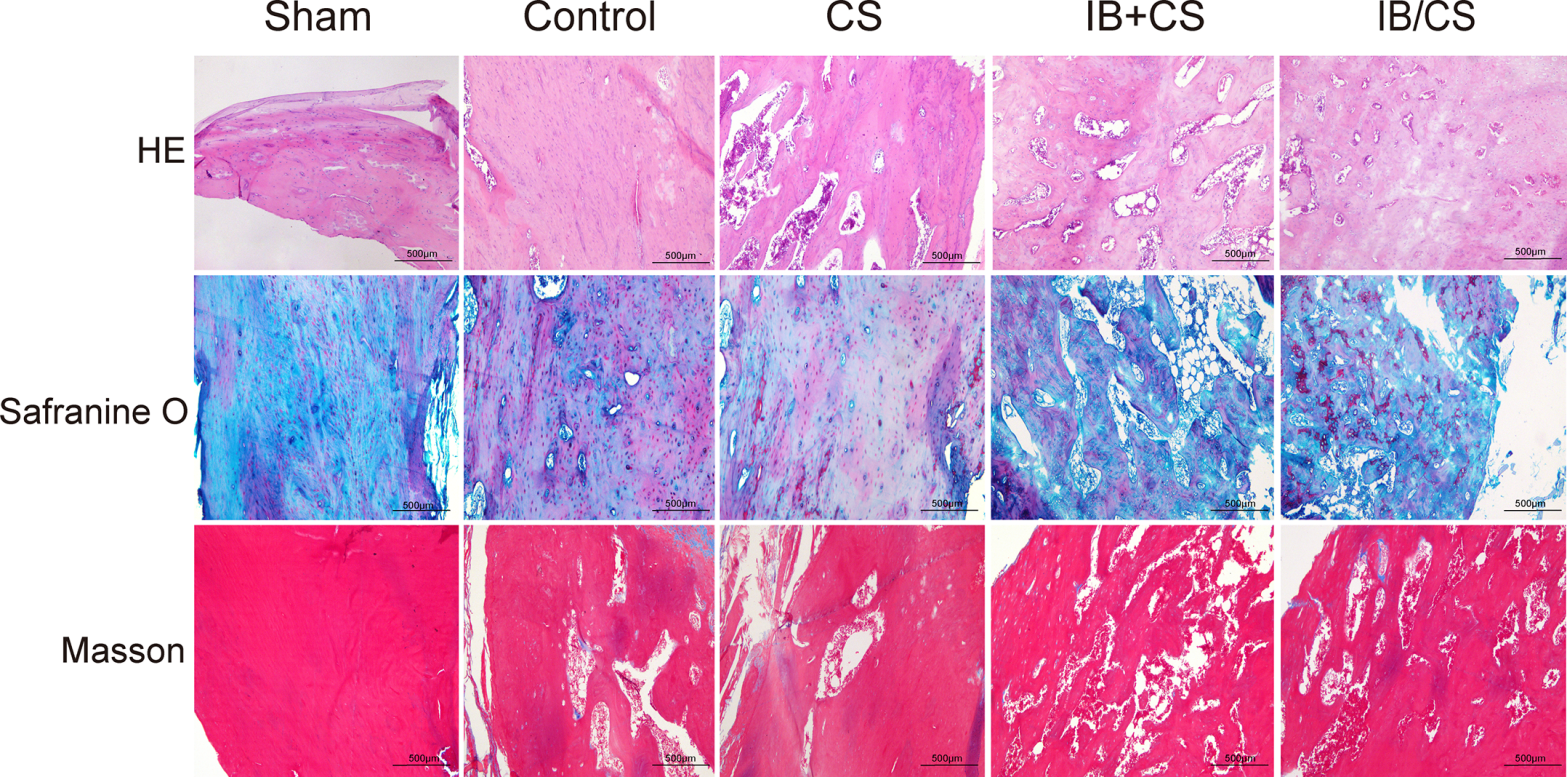
Histological evaluation of the fracture in each group at 12 weeks after surgery. HE staining. Safranin O and fast green staining, Masson staining. Scale bar = 500μm.

The callus was regularly compacted with a lamellar conformation in the Sham group 12 weeks after fracture fixation. Abundant compacted lamellar bone and a small amount of woven bone were observed in the Control and CS groups. There was a significant decrease in the amount of remodeled woven bone with alignment to the longitudinal direction in the IB treatment groups, and primary lamellar bone was found. The differences in fracture callus content of the un-remodeled woven bone were not obvious in the CS+IB and CS/IB groups (Figs 6 and 7).

### Safranin O/light green-staining

Most sites in the Sham group were occupied by woven bone at 4 weeks. Conversely, larger areas of cartilage, which was demonstrated in the Control group, indicated that open fracture repair proceeded through an endochondral ossification process that was much slower than those in the Sham groups. Compared with the Control group, there was an obvious increase in primary bone in the CS. In the IB treatment groups, the process of remodeling to the irregular woven bone was earlier and active, but the fibrocartilage still existed. The callus in CS/IB was remarkably larger than the CS group. At 12 weeks, the callus in the Sham and Control groups showed fully compacted lamellar bone, which was aligned to the longitudinal axis of the femur. Although partially compacted lamellar bone with an oriented parallel array was observed, parts of the callus consisted of woven bone in the CS+IB and CS/IB (Figs 6 and 7).

### Masson staining

More than half of the amount of blue-stained bone tissue (immature) was found in the Sham group at 4 weeks. In the control group, most parts in the fracture healing zone were composed of connective tissues with a small amount of blue dye. Compared with the Control group, abundant vascular activity was observed within the more newly formed woven bone in the CS group. Masson’s trichrome staining results indicated that IB treatment was beneficial for new bone formation. More newly formed blue-stained callus was observed around the fracture site in the IB/CS.

A large amount of the red-colored region (mature) was observed in the Sham group at 12 weeks, and most woven bone was replaced by compacted bone. In the Control and CS group, red-colored tissue was observed but was smaller than that in the Sham group. The majority of the woven bone had calcified and had been remodeled into lamellar bone. In IB treatment groups, more immature red-colored structure, which was a sign of the normal bone remodeling process, was found due to inhibition of remodeling, but there were no significant differences in the CS+IB and CS/IB groups (Figs 6 and 7).

Furthermore, quantitative comparisons of the dynamic histomorphometric parameters demonstrated that the Control group showed significantly lower MAR and BFR/BS than the Sham group at 4 and 12 weeks. In comparison with the Control group, rats in the CS+IB and CS/IB groups exhibited significantly increased MAR and BFR/BS at 4 weeks post injection of sustained-release drugs. Although the lamellar bone remodeling process was delayed by IB treatment at 12 weeks, the larger callus was able to provide stability to the fracture (Table 1).

**Table 1:** Effects of local drug sustained-release delivery (including IB) on trabecular bone histomorphometry in rats subjected to ovariectomy combined with femoral fracture.

Bone histomorphometry in fracture callus at 4 weeks post-fracture					
Group	Sham	Control	CS	CS+IB	CS/IB
Ca.Ar (mm^2^)	7.2±0.9	11.4±1.9*	13.3±1.3^a^	14.6±1.1^a^^,^^b^	16.7±1.3^a^^,^^b^^,^^c^
Lamellar/Ca.Ar (%)	45.3±3.0	12.2±2.1*	17.5±3.1	9.8±2.0^a^^,^^b^	8.3±1.2^a^^,^^b^
BFR/BS (μm3/μm2 per day)	0.98±0.12	0.54±0.13*	0.74±0.14	1.13±0.11^a^^,^^b^	1.37±0.11^a^^,^^b^
MAR (μm/day)	1.71±0.19	1.31±0.11*	1.41±0.13	1.61±0.17 ^a^^,^^b^	1.88±0.14 ^a^^,^^b^^,^^c^
Bone histomorphometry in fracture callus at 12 weeks post-fracture					
Group	Sham	Control	CS	CS+IB	CS/IB
Ca.Ar (mm^2^)	9.4±1.2	8.1±1.1*	8.2±1.3	11.3±2.1^a^^,^^b^	13.6±1.7^a^^,^^b^
Lamellar/Ca.Ar (%)	97.5±0.7	97.8±0.94*	98.6±0.89	80.9±1.13^a^^,^^b^	78. 2±3.9^a^^,^^b^
BFR/BS (μm^3^/μm^2^ per day)	0.74±0.09	0.43±0.13*	0.47±0.24	0.39±0.18	0.47±0.11
MAR (μm/day)	1.61±0.11	1.25±0.09*	1.27±0.13	1.05±0. 17	1.01±0. 34

Values represent the mean ± SD. Each group was formed by 6 rats.

*p<0.05 compared to Sham.

^a^ p<0.05 compared to Control.

^b^ p<0.05 compared to CS

^c^ p<0.05 compared to CS+IB.

## Discussion

The primary aim of this study was to validate that a local applied collagen sponge combined with IB can accelerate femoral fracture healing in ovariectomized rats. To avoid the limitation of systemic therapies, several studies highlighted the excellent efficiency of local delivery of drugs and proteins in repairing fractures and improving implant surface osseointegration [9,16], whereas the effect of local drug on osteoporotic fractures is still poorly understood. Local delivery is appealing because it avoids adverse effects found in systemic administration and minimizes the required dose of drugs. This investigation showed that collagen sponges combined with IB as a delivery system was an excellent option and effective at improving fracture healing in an osteoporotic rat model. Compared to systemic administration, it can improve the quality and quantity of the callus by changing microscopic structures of the callus and improving histological parameters and mechanical properties of the callus. The relatively large amount of woven bone observed at the early stage is attributed to the aggregative effect from CS on chondrocytes, fibroblasts and the potent suppressed effect of IB on osteoclasts [1,10]. This research enriches our understanding of local drug delivery and provides a definite therapeutic effect on osteoporotic fractures.

Fracture healing is complicated and characterized by four well-orchestrated stages that are initiated as a response to injury. With an open osteotomy in our research, the periosteum is seriously damaged due to soft tissue stripping involved in the closed fracture model, and endochondral ossifications play a more important role in femoral fracture healing. The semi-rigid fibrocartilage callus (soft callus) formed from hematoma provides initial stability at the early stage of fracture union. The largest volume of soft callus and a certain amount of woven bone were observed and attributed to improvement of the mechanical properties in the CS/IB group at 4 weeks. The final stage of fracture healing is dominated by the remodeling process of woven bone affected by osteoclast activity. Some of the primary mineralized soft callus is gradually changed and remodeled into lamellar bone or spongiosa depending on the location where fracture repair happens. In the Control and CS groups, histology revealed that the majority of woven bone had been converted into compacted lamellar bone at 12 weeks. However, the callus in the CS+IB and CS/IB groups consisted of small lamellar and more woven bone undergoing active remodeling. Generally, mineralization of fibrocartilage/cartilage was almost completed at 4 weeks in normal rats with an open osteotomy [17]. In the osteoporotic rat model induced by ovariectomy and employed in our study, there is a negative balance and high turnover state in bone formation and resorptions, and the remodeling process of fibrocartilage/cartilage is delayed and remains at 6 weeks due to estrogen deficiency [18,19,20]. In accordance with pathogenesis, the decreased volume of callus, deterioration of trabecular bone and reduction of mineralization were confirmed by the decrease in mechanical strength and microarchitecture in the Control group compared with the Sham group at 4 weeks and 12 weeks by μCT scans, mechanical tests and histopathology [21]. High serum levels of PINP and CTX indicate that ovariectomized rats have a high bone turnover rate. Based on the above, the pathological state of osteoporosis along with microarchitecture damage, excess inflammatory signaling and mechanical instability seriously impair fracture healing at the early stage of osteoporotic fracture. Therefore, early interventions such as drugs or other anti-resorptive agents are beneficial and necessary to prevent the occurrence of nonunions in osteoporotic fractures. We will mainly focus on drug therapy (especially IB) to fractures.

Medical therapies of fracture encompass anabolic and catabolic agents. Parathyroid hormone (PTH) and pertussistoxin (PTX) act on osteoblasts by the PTH/PTHrP receptor 1 (PTHR1) and G5 signaling pathway to prevent bone loss and increase bone remodeling [22]. However, the increase in bone remodeling may lead to unmaturation of collagen fibers and weak bone quality. As an anti-resorptive drug, IB exerts its anti-bone resorptive effect by inhibiting the attachment of osteoclasts to the bone matrix and inducing apoptosis action to regulate the balance between osteoclast formation and bone resorption in fracture healing [1,23,24,25]. Then, the catabolism of newly formed woven bone is regulated and suppressed by BPs, and the non-union rate is decreased^12^. Other disruptive effects are that BPs have a different potency to prevent the formation of osteoclast-like cells, which was also proven by Hughes et al [26]. However, there is a debate about the most optimal model of delivery with BPs to improve the mechanical strength of the fracture [27,28]. Most of them focus on the time, different doses and treatment schedules to evaluate the effects on fractures [17,29,30]. There is not a unanimous conclusion from these studies, and the differences in drugs, animal species and different endpoint times explain the diversities among the conclusions. A local delivery system is another choice for increasing the availability of IB and shortening the healing period. The local delivery of BPs contains implant coatings, bone cement and intraosseous administration [21,31,32]. Gao et al compared three BPs incorporated into hydroxyapatite-coated implants and showed improved osseointegration or push-out mechanical strength after 3 months in the tibia of ovariectomized (OVX) rats [8]. Compared with other BPs, IB had a relatively gentle release from the hydroxyapatite surface through a release test [33]. Consistent with these studies, cartilage and fibrocartilage in the CS/IB were significantly decreased and more woven bone, which provided the initial stability and mechanical function, was formed at 4 weeks, whereas there was a large amount of fibrocartilage in the Control group. This confirmed the inhibited effect of IB on osteoclasts. We found that IB improved the structural characteristics of bone tissue, which was confirmed from the improvement of BV/TV, the density of BT, the density of TV, Tb.N, Tb.Th and connectivity parameters and the decrease in Tb.Sp by Micro-CT evaluations. In accordance with the above results, local applied IB provided better mechanical performance than the Control, CS and CS+IB groups. The improvement in structural characteristics seems to contradict the mechanism of IB, and it is traditionally believed that BPs maintain bone microarchitecture rather than cause an improvement, but more and more studies suggest that there is a positive effect on bone [18,34,35]. Amanat et al found that a single local delivery of pamidronate could increase callus volume, but the mechanical strength was not improved and systemic exposure still existed in the contralateral limb [12]. However, we noticed that when a poly (D, L-lactide) (PDLA) coating incorporated with pamidronate was used, the release rate of drugs is inferior to collagen. Conversely, Hao et al reported that there was an obvious enhancement of bone microarchitecture after treating with zoledronic acid compared with that of OVX rats in an open fracture model and explained the true reason, which was relevant to zoledronic acid^18^. Since more and more evidence suggests that the osteoclast is somewhat redundant in remodeling of fibrocartilage callus, the reasons for the improvement of CS/IB in 4 weeks are considered to be due to two reasons. First, a higher concentration of IB in CS/IB increases calcification of remodeled bones on their surfaces and new bone deposits in the excavated sites formed by osteoclasts. Second, the slowdown rate of remodeling and catabolism to immature newly formed bone increases the accumulation of woven bone and the time of mineralization, and the negative balance may be changed and even reversed at certain times during IB treatment. Besides the inhibition of osteoclasts, some studies report that BPs can also increase the differentiation of osteoblasts [36,37]. Our results showed that there was a significant increase in callus formation in the CS+IB compared with that of the CS at 4 weeks. Although the delayed remodeling of the bone callus was observed in the CS+IB and CS/IB groups, the mechanical strength was still improved at 12 weeks. Meanwhile, we noticed that there was an improvement of the microarchitecture in the CS group and concluded that the enhancements of CS/IB were also attributed to the CS in addition to IB. In a previous study, the local use of IB improved ossification of distal femoral osteotomy in a rabbit model at the early stage of bone union [7]. Our present study with osteoporotic fracture, which employed CS as the carrier, is more convincing than regular fracture. Besides the positive effect of IB on fracture healing, more concerns should also be raised to investigate the effect of the carrier on fracture healing.

Compared with catabolic action, more attention has been paid to the anabolic response when treated with substitute drugs, but it is rare to find reports that focused on the anabolic effect of the delivery system. Many biomaterials are employed as local applied scaffolds, such as polyglycolic acid (PGA), poly(lactic-co-glycolic acid) (PLGA), polycaprolactone and fibrin, but there are a number of disadvantages such as low porosity and toxic degradation [38]. CS is an ideal carrier for drug delivery and is widely used in hemostasis of skin wounds and for filling and repairing the residual tissue cavity. It is employed to change the surface feature of other scaffolds to improve their biodegradability, biocompatibility, hydrophilicity and hemostasis properties [39].

CS is the main component of bone and is stiffened by calcium hydroxyapatite; therefore, it is an excellent choice as the basis for local delivery that is capable of supporting and promoting the fracture union process. Local application of CS as a carrier focuses the drug to the osteotomy site and minimizes the unnecessary biodistribution of IB to other tissues compared with oral or systemic administration [40]. Besides these characteristics, as a scaffold, it provides an ideal biophysical environment that is beneficial for tissue regeneration [41]. With loosened and porous structures, CS serves as a scaffold or anchorage that facilitates cell migration, adhesion and infiltration, provides oxygen and nutrients, and excludes waste from cells [13,42]. In fracture healing when combined with other bone particles such as demineralized allogenic bone matrices or BMP through infiltrating and diffusing, it promotes the formation of loose connective tissue around the fracture and differentiation of mesenchymal cells into osteogenic cells [43,44]. Horisaka et al used the CS/BMP plant to study its osteogenesis and observed that the pores of CS facilitated the migration of many cells [45]. CS accelerates and improves the formation and organization of the hematoma after fracture, and therefore, it is beneficial to the repair of the bone defect through enlarging the quantity of fibrous tissue at an early stage [46]. Gleeson et al developed a CS incorporated with hydroxyapatite particles and found the formation of mineralized tissue with transosseous critical sized defects in the collagen-only scaffold [47]. Consistent with these reports, a larger amount of woven bone was observed in the CS than in the Control group, and sufficient woven matrix was beneficial to the formation of lamellar bone on its cores [48]. However, the microarchitecture was not changed compared with the Control groups. The main mechanism for improving the ultimate load of bone is an increase in bone volume, especially fibrocartilage at an early stage. The hematoma of the CS group is larger at the early stage of fracture healing, which is attributed to the water uptake character of collagen. Vascularization is essential for fibrocartilage callus (soft callus) remodeling to woven bone (hard callus) via intramembranous and endochondral ossification at the osteotomy site. The scaffold structure of CS provides a beneficial environment for subsequent vascularization and cell proliferation and ingrowth, differentiation and mineralization of osteoblasts, which synthesize initial woven bone [49, 50]. The vasculature is proven by the appearance of small blood vessels or capillaries at the early stage of collagen implantation^43^. Besides, it provides a physical surface for IB, and IB is released passively in the early stage and then is gradually and actively diffused along with the degradation of CS to avoid unnecessary effects such as ectopic ossification caused by uncontrolled delivery [51]. However, it is traditionally suggested that CS is not preferable for bone tissue engineering due to its poor load-bearing capabilities, easily degraded structures and low anti-tension ability. Therefore, the product combined with bisphosphonates is utilized in our research to offset the disadvantage of CS and is crosslinked to improve the mechanical properties through chemistry methods. The CS degradation rate can be adjusted through manipulation of the crosslinking degree or by adding fibrinogen and thrombin to improve the properties of CS [38]. However, considering the negative effect of ibandronate on bone remodeling with late administration and optimizing the delivery of IB through controlled short-term release, other changes that stabilize the structures of CS were not tested [18]. Above all, CS has the potential to be employed as an excellent local delivery for short-term controlled release of IB.

The treatment of osteoporotic fractures is always a challenging job because of the frequent occurrence of delayed healing. The fracture healing process of OVX rats is seriously impaired in the early phase. The remodeling of fibrocartilage callus to woven bone was not completed at 4 weeks in the Control group, and the cartilage was much more easily observed compared with Sham. Just as we mentioned above, IB treatment cannot affect the remodeling process of fibrocartilage, but it acts through two ways to stimulate the accumulation of woven bone and prolong the subsequent time of mineralization. There were large amounts of woven bone in the CS/IB group compared with CS+IB at 4 weeks. Additionally, the microarchitecture and mechanical strength of CS+IB were lower than those in CS/IB, but almost same amount of hard callus was remodeled into lamellar bone at 12 weeks. After excluding the effective biodistribution of drugs at the local site with local delivery, an explanation for the increase in mechanical strength and microarchitecture in the CS/IB group is attributed to the synergistic effects between collagen and IB on fracture healing. Research by Sakai et al indicated that BPs administered with eldecalcitol showed a synergistic effect on the inhibition of bone resorptions [52]. The anabolic ability of collagen combines perfectly with the catabolic character of IB, and it is concentrated on the fracture site to improve osteoporotic fracture healing with the combined effect on the activity of osteoblasts and osteoclasts. There are three explanations for the improvement in CS/IB. First, the product makes controlled short-term release related to the degradation of collagen possible. Manabe reported that a larger callus was observed by daily administration of IB compared with single dose administration [14], and the product mimics daily administration and improves callus formation as much as possible. Second, it is also reported that IB binds to bone matrix that existed at the time of injection; however, the best timing of administration is still under debate. Hao reported that the earlier the administration, the more positive was the result, but Amanta considered that a delayed single dose resulted in a larger callus and better mechanical strength^17^. We understand that bone catabolism, which lags behind anabolism, is not absolutely active after the first day of fracture, and systemic administration may lead to a decreased effect when IB is combined with inactive bone matrix [53]. The sustained controlled delivery can lose its stability, and the degradation of collagen results in a negative release that turns into an absolute release; this is beneficial to IB combined with active osteoclasts occupying the bone surface. Boerckel et al studied the delivery of BMP with the collagen sponge and concluded that the half-life release was 1.87 days and that most of the BMP delivered was released in the early days [54]. Gisela evaluated the character of mesoporous silica nanoparticles as a local delivery for IB and found that pure collagen had fast release kinetics and that 80% of IB was released in the first 2 days in vitro [55]. In our study, collagen was degraded and absorbed completely 4 weeks after femoral fracture. A greater amount of new bone that is formed at the early stage is maintained with an effective concentration of IB, and this is consistent with the result that there is a more impressive increase in bone callus in the CS/IB group compared with that of systemic administration at 4 weeks. However, the inhibition of remodeling in CS/IB had no significant differences compared with CS+IB, and the mechanical strength was increased by sufficient callus in CS/IB inversely at 12 weeks. This is explained by the fact that IB is mainly incorporated with relatively early formed bone, and the suppression of callus remodeling is reduced because bone formed later is devoid of IB. The bone remodeling time in OVX rats is reported to be approximately 13–24 days [29], and there is sufficient time for the accomplishment of remodeling. It is important to note that the dose required in CS/IB (0.04 mg) is lower than that in the CS+IB group (0.015 mg/100 mg, the ovariectomized rats were all larger than 300 mg after 6 months in our research). Moreover, another advantage of this product is that the treatment is simplified, as drug delivery and operations are integrated together in one procedure. Besides, the adopted osteoporotic model is also an important reason for the more pronounced effect on bone resorption. On the one hand, the osteoporotic trabeculae acts as a quasi-scaffold for the formation or deposition of woven bone and provides a wider space for the drugs to take effect compared with normal bone tissue in the Sham group. On the other hand, the high turnover rate of local bone around osteoporotic fracture increases the release of IB from bone [52]. Besides the early release of drugs, another reason for minimized inhibition of bone remodeling is the lower binding affinities, different pharmacokinetics and reduced persistence of the effect for hydroxyapatite than that of zoledronate [17]. Interestingly, rats studied soon after menopause are more inclined to recover from the bisphosphonate therapy. In addition, the different concentrations of IB may have effects on regulating bone quantity and bone microarchitecture. The limitation of this research is as follows: first, compared with data for the release kinetics in vitro, although collagenase was added to mimic the status in vivo, the intrinsic variations in rats were more complicated. Thus, more research is needed to explore the best concentration and provide valuable support for local IB delivery based on collagen to treat osteoporotic fractures. Second, the experiment duration last for 12 weeks, and an experiment lasting for more than 12 weeks until the remodel phase completed will be conducted to test the biomechanical properties in IB treatment groups in future.

## Conclusion

In conclusion, this is the first report demonstrating the short- and long-term effects of local IB combined with collagen on bone microarchitecture, mechanical properties and histology in osteoporotic fractures. As a local controlled short-term release delivery, CS combined with IB exhibits a synergistic effect and exerts a promotional effect, which is superior to systemic administration of IB. It demonstrates that open fracture is stabilized at the early stage of endochondral ossification and does not reduce the callus quality at the end of the healing process in an osteoporotic fracture. The suppression of remodeling is comparative with systemic administration, and the structurally less effective bone strength produced by woven bone formation is compensated for by a larger callus volume with the local delivery system. Our study focuses on the effect of local drug delivery to improve osteoporotic fracture healing, and local applied CS/IB results in earlier, faster and more efficient protection from osteoporosis-associated fractures, especially in the early stage of healing. However, more work is needed to enhance the stability of the collagen scaffold on the premise of reducing its suppression effect on bone remodeling. Above all, we provide a feasible method using a collagen sponge combined with IB to treat femur fractures.
